# Incidence, Mortality, and Predictive Factors of Hepatocellular Carcinoma in Primary Biliary Cirrhosis

**DOI:** 10.1155/2013/168012

**Published:** 2013-02-25

**Authors:** Kenichi Hosonuma, Ken Sato, Masatoshi Yanagisawa, Satoru Kakizaki, Hitoshi Takagi, Junko Hirato, Masatomo Mori

**Affiliations:** ^1^Department of Medicine and Molecular Science, Gunma University Graduate School of Medicine, 3-39-22 Showa-machi, Maebashi, Gunma 371-8511, Japan; ^2^Department of Pathology, Gunma University Hospital, 3-39-15 Showa-machi, Maebashi, Gunma 371-8511, Japan

## Abstract

*Background*. The study aims to analyze in detail the incidence, mortality using the standardized incidence ratio (SIR), and standardized mortality ratio (SMR) of hepatocellular carcinoma (HCC) in primary biliary cirrhosis (PBC), because no large case studies have focused on the detailed statistical analysis of them in Asia. *Methods*. The study cohorts were consecutively diagnosed at Gunma University and its affiliated hospitals. Age- or sex-specific annual cancer incidence and deaths were obtained from Japanese Cancer Registry and Death Registry as a reference for the comparison of SIR or SMR of HCC. Moreover, univariate analyses and multivariate analyses were performed to clarify predictive factors for the incidence of HCC. *Results*. The overall 179 patients were followed up for a median of 97 months. HCC had developed in 13 cases. SIR for HCC was 11.6 (95% confidence interval (CI), 6.2–19.8) and SMR for HCC was 11.2 (95% CI, 5.4–20.6) in overall patients. The serum albumin levels were a predictive factor for the incidence of HCC in overall patients. *Conclusions*. The incidence and mortality of HCC in PBC patients were significantly higher than those in Japanese general population. PBC patients with low serum albumin levels were populations at high risk for HCC.

## 1. Introduction

Hepatocellular carcinoma (HCC) has been increasingly reported as a complication of primary biliary cirrhosis (PBC) [1–9]. Large case studies showed that the incidence of HCC [1, 2, 8, 9] in patients with PBC was significantly higher than those in the general population. However, the incidence of malignancies including HCC is still controversial [10]. On the other hand, only a few studies [2, 4, 11] reported predictive factors for the incidence of HCC in PBC. Most of the large studies about the incidence of HCC have been performed in Europe and America, and no large case studies have focused on the detailed statistical analysis of the incidence and mortality using standardized incidence ratio (SIR) and standardized mortality ratio (SMR) of HCC in PBC in Asia. Here, we evaluated the incidence, mortality, and predictive factors for the incidence of HCC in PBC in Japan.

## 2. Patients and Methods

The present study included the patients who were consecutively diagnosed at Gunma University and its affiliated hospitals as having PBC between 1988 and 2011, and who were followed up for more than or equal to 12 months. In addition, the eligibility of the cohorts is that HCC screening interval is 6 to 12 months. We reviewed the medical charts retrospectively to evaluate these subjects and extracted patients who had developed HCC from the time of the diagnosis of PBC to the date of death, or on September 30, 2011. The study protocol was compliant to the ethical guidelines of the 1975 Declaration of Helsinki.

The criteria for the diagnosis of PBC were positive antimitochondrial antibody test assessed by enzyme-linked immunosorbent assay in combination with increased serum alkaline phosphatase activity and compatible liver histology. We confirmed histological assessment at diagnosis of PBC in all subjects. Histological findings were classified according to Scheuer's classification [12]. Symptoms of PBC were defined as pruritus, persistent fatigue (lasting greater than three months in the absence of other identifiable causes such as anemia or hypothyroidism), overt jaundice, bleeding esophageal varices, ascites, hepatic encephalopathy or persistent right hypochondrial pain in the absence of another cause such as cholelithiasis [13].

HCC was diagnosed if the following conditions were met: (1) pathological features consistent with HCC were identified by histological examination of liver tissue obtained by needle biopsy or other procedures; or (2) one or more hepatic space-occupying lesions were detected by ultrasonography or computed tomography and were shown to have vascular patterns typical of HCC by angiography, triple-phase spiral computed tomography, or contrast-enhanced magnetic resonance imaging. Because our study was performed using several modalities to screen HCC and the screening interval is 6 to 12 months, we assessed the comparability of the ratio of types of modalities to screen HCC, the screening intervals and the ratio of combination of modalities, and the screening intervals between the patients who had developed HCC and those who had not developed HCC. On the other hand, ursodeoxycholic acid improves prognosis in view of liver biochemistry, histological progression, the development of portal hypertension and its complications [14–18], and combination of ursodeoxycholic acid and bezafibrate [19–21] or bezafibrate alone [21, 22] are effective for PBC. Moreover, several observational studies [18, 23–27] have shown that the long-term outcome of PBC correlates with the biochemical response to ursodeoxycholic acid. Then, we evaluated the ratio of types of therapeutic regimen and the ratio of groups with responders or non-responders to ursodeoxycholic acid by the criteria based on Barcelona [24], Paris [25], Rotterdam [26], and Toronto definitions [27], patients who received only bezafibrate or those who received neither ursodeoxycholic acid nor bezafibrate between those who had developed HCC and those who had not developed HCC. Time to event was calculated by the intervals from the diagnosis of PBC to the diagnosis of HCC. Each principle physician censored the development of HCC. The analyses have been carried out with or without the period of follow-up for one year after the diagnosis of PBC because of reducing the impact of selection bias (the investigation of symptoms shortly after the diagnosis of PBC might increase the chance of cancer being investigated and diagnosed). Person years at the risk of follow-up in the incidence and mortality were calculated for each patient starting immediately (and one year) after the diagnosis of PBC, and ending with HCC registration, death, or end of the follow-up period. Age- or sex-specific annual cancer incidents and deaths were obtained from Japanese Cancer Registry and Death Registry. The SIR or SMR and their 95% confidence intervals (CI) based on the Poisson distribution were then calculated using the population of the whole Japan as the standard population because these data were not available in the Gunma prefecture.

All data about these parameters were collected in the medical charts, death certificate, and questionnaires. The predictive variables of the development of HCC were extracted from the data at the diagnosis of PBC. Although some data was missing, definitive histological diagnosis of PBC and complying with screening methods for HCC was requisite for registering as our study cohort. The analysis about SIR, SMR, and predictive factors was conducted for overall or female patients because the number of males was small. We assessed the associations between the incidence of HCC and laboratory and clinical variables including several variables (cigarette smoking; alcohol consumption; blood transfusion; familial history of malignancies; liver diseases; autoimmune conditions; diabetes mellitus; hypertension; hyperlipidemia, and body mass index). Continuous data are presented as median and range. For quantitive data, analyses were performed using the Mann-Whitney *U*-test for comparison of two independent groups. Differences in proportions were performed by the Fisher's exact test. The Spearman's rank-correlation coefficient (two-tailed) was used to evaluate the correlation between the variables. Basically, variables that achieved statistical significance and marginal significance on univariate analysis were entered into multivariate analysis by the Cox proportional hazards model to identify significant independent predictive factors. *P* < 0.05 was considered significant. All analyses were performed using the IBM SPSS statistical software package, version 16 (IBM Corp., Armonk, NY, USA).

## 3. Results

The clinicopathological characteristics of the study population at diagnosis of PBC were summarized in Table 1. The overall sample included 179 patients (24 males, 155 females). The follow-up period ranged from 12 to 281 months with a median of 97 months. The median age was 57 years (range 22–85). We completed follow-up of the patients without any dropout. One hundred and one patients had Scheuer's stage I, 42 had stage II, 19 had stage III, and 17 had stage IV. Of a total of 179 patients, 53 (29.6%) were symptomatic at diagnosis. Anti-HCV antibody and hepatitis B virus surface antigen were tested in all patients. No patients were positive for anti-HCV antibody and hepatitis B virus surface antigen. Although hepatitis B virus core antibody was not measured in some patients, all patients who had developed HCC were negative for the antibody. The ratio of types of the modalities to screen HCC and the follow-up period were not significantly different between the patients who had developed HCC and those who had not developed HCC in overall or female patients (Table 2). In addition, the ratio of combination of the modalities and the follow-up period such as biannual CT were not significantly different between them in overall or female patients. Furthermore regarding the types of therapeutic regimen and the ratio of groups with responders or non-responders to ursodeoxycholic acid defined by the criteria as mentioned above [24–27], patients received only bezafibrate or those who received neither ursodeoxycholic acid nor bezafibrate were not significantly different between those who had developed HCC and those who had not developed HCC in overall or female patients (Table 2).

During follow-up, HCC had developed in 13 cases (Table 2). The median age of the patients at the diagnosis of HCC was 68 years (range, 56–80). The median age of the patients at the diagnosis of PBC was 63 years (range, 46–77). The median period from the time of the diagnosis of PBC to the time of the diagnosis of HCC was 76 months (range, 9–180). All of the HCC was diagnosed through screening alone, but not a workup of new symptoms. The diameter of all detected HCC is less than or equal to 3 cm. Of 13 HCC cases, histological examination of the background liver was performed in five cases and showed that one had Scheuer's I, one had Scheuer's II, one had Scheuer's III, and two had Scheuer's IV. Five cases had TNM stage I, six had stage II, and two had stage III at the HCC diagnosis. As for the treatments, operation, transcatheter arterial chemoembolization, and/or radiofrequency ablation and microwave coagulation therapy could be applied to ten cases, and three cases were incurable because of their poor liver function.

The total number of person years at the risk of follow-up in the incidence calculation was 1,608 when using all incident HCC and 1,447 when omitting the first year of follow-up after PBC diagnosis in overall patients.  Table 3 shows the SIR of HCC. The SIR of HCC in overall or female patients was significantly higher than that in general population whether the first year of follow-up after PBC diagnosis was excluded or not.

The total number of person years at the risk of follow-up in the mortality calculation was 1,776 when including all deaths. The length of follow-up for subjects varied between 12 and 281 months with a median of 97 months. The primary cause of death includes 13 cases of malignancy death (nine cases of HCC, one case of lung cancer, one case of malignant lymphoma, one case of uterine corpus cancer, and one case of skin cancer), nine cases of liver failure, and nine cases of other causes such as subarachnoid hemorrhage (Table 4).  Table 3 gives the SMR of HCC. The SMR of HCC in overall or female patients was significantly higher than that in general population.

The overall prevalence of associated autoimmune conditions was 32.4%. Sjögren's Syndrome was the most frequent association: it was present in 14.5% of cases. Thyroid disease was observed in 8.9% and rheumatoid arthritis in 8.9% of cases. Systemic sclerosis was recorded in 8.4% and systemic lupus erythematosus in 0.6% of cases.

A history of smoking (more than 10 cigarettes per day) was observed in 20.7% (14.8% in females, 58.3% in males). A history of alcohol consumption more than 20 g/day in males was 16.7% and that more than 20 g/day in females was 4.5%. The rate of obesity (body mass index; 30 or more) was 1.7%. A history of blood transfusion before a PBC diagnosis was observed in 12.8% of cases. A familial history of malignancy was observed in 35.8% and familial history of liver disease in 14.5% of cases. Diabetes mellitus was observed in 10.6% of cases, hypertension in 17.9%, and hyperlipidemia in 24.0% of cases.

Variables that associated with the development of HCC were analyzed by both univariate and multivariate analyses. A univariate analysis identified some variables associated with the development of HCC in overall or female patients, which reached statistical significance or marginal significance. Among these variables, however, some variables were significantly correlated with a certain variable or others by the Spearman's rank-correlation coefficient. Then we used variables that were not significantly correlated one another. In the analysis of overall patients, these variables included Brinkman index (number of cigarettes per day × years of smoking), the serum albumin levels, the serum AST levels, the platelet counts, and the past history of Hashimoto's disease. A multivariate analysis identified the serum albumin levels that independently influenced the development of HCC in overall patients (Table 5). In the analysis of female patients, variables identified by the same method as mentioned above included Brinkman index, the serum albumin levels, the serum AST levels, the platelet counts, and the past history of Hashimoto's disease. A multivariate analysis identified the serum albumin levels that independently influenced the development of HCC in female patients (Table 5).

## 4. Discussion

In our study, the incidence risk of HCC was significantly increased in PBC patients in comparison to adjusted general population with or without the period of follow-up for a year after PBC diagnosis. The increased incidence risk of HCC in PBC patients was consistent with the previous crude large case study in Japan [28]. Our study is of quite value in view of the precise analysis using SIR and SMR differing from the previous study [28]. Outside Japan, the increased incidence risk of HCC in PBC patients is controversial [1, 2, 8–10]. Loof et al. [10] examined 559 patients with PBC with a mean follow-up period of 9 years in Sweden; they concluded that there was no significant increase in the occurrence of HCC in patients with PBC. However, HCC may have remained undetected in this study, because less than 50% of the deceased patients had an autopsy performed. In addition, racial difference may contribute to the incidence of HCC in PBC patients.

Among the large population-based studies, only a few studies [2, 4, 11] examined the predictive factors for the development of HCC. Reported predictive factors were cigarette smoking [2], HCV positivity [2], older age at diagnosis [4], male sex [4], history of blood transfusion [4], and advanced histological stage [11]. The serum albumin levels were the independent predictive factor for the development of HCC and significantly correlated with symptom of PBC, histological stage at the diagnosis of PBC, or age at the diagnosis of PBC by the Spearman's rank-correlation coefficient in our study. In addition, symptomatic PBC frequently shows advanced stage of PBC [29]. Taken together, the serum albumin levels considered as an indicator of advanced PBC were selected for the predictive variable of HCC, supporting the recent study [11]. On the other hand, branched-chain amino acid is reported to increase serum albumin levels in patients with decompensated cirrhosis [30] and inhibits hepatocarcinogeneis in patients with decompensated cirrhosis with higher baseline body mass index or alpha-fetoprotein levels [31] and with compensated cirrhosis with lower baseline albumin levels [32]. In cirrhotic patients, total albumin levels decrease and simultaneously a ratio of oxidized albumin within total albumin increases. Antioxidant properties of oxidized albumin is reportedly weaker than those of reduced albumin [33]. Although being speculative, reduced antioxidant effect based on hypoalbuminemia and concomitant change of quality of albumin in advanced stage of PBC might be one of the reasons of hepatocarcinogensis.

Because our study includes small sample size of male study cohorts, the interpretation of contribution of male sex to incidence of HCC is limited. In our study, a history of smoking was not a significant predictive factor for the development of HCC, although relatively higher cigarette consumption in female study cohorts was found, suggesting that racial differences of the study population between the study [2] and the other studies [4, 11] including our study may be one possible reason of the difference of the predictive factors. The history of blood transfusion in our study was 12.8%, comparable to 15.6% of that in a total of 119,792 study cohorts in The Japan Collaborate Cohort Study for Evaluation of Cancer Risk (JACC Study) [34] and 11.6% in the previous study [4] that identifies the history of blood transfusion as a predictive factor for HCC development. Thus, although the exact reasons of the difference of the predictive factors between the study [4] and our study are unknown, the possible reason is that it might affect carcinogenesis by infections with occult hepatitis B virus or unknown transfusion-transmissible viruses in the earlier study [4] due to a hospital-based study.

Malignancy death was the most common and HCC occupied the bulk of malignancy death in our study. Our study found evidence for an excess mortality from HCC (SMR, 11.2; 95% CI, 5.4–20.6), which confirms the studies in The Netherlands [35] and England [1].

The limitation of our study is that the sample size of the study cohort is relatively small, our study population derived from the hospitals may increase the SIR and the SMR, and our study population based on a local Japan area may not always reflect data derived from a nationwide register.

## 5. Conclusion

In conclusion, our detailed analysis showed that the incidence and mortality of HCC in overall PBC patients were significantly higher than those in Japanese general population. PBC patients with low serum albumin levels were populations at high risk for HCC. Although therapeutic options in HCC patients with low serum albumin levels may be limited, early detection of HCC may result in inhibition of reduction of the therapeutic options and thus improve the prognosis in some patients. Thus, regular monitoring for early detection of HCC may be a good strategy for PBC patients with low serum albumin levels.

## Figures and Tables

**Table 1:** Clinicopathological characteristics of the study population.

Parameters	Male	Female	Overall
Number of patients	24	155	179
Age of diagnosis of primary biliary cirrhosis (years)*	55.5 (22–75)	57 (24–85)	57 (22–85)
Duration of follow-up (months)^∗†^	96.5 (27–218)	97 (12–281)	97 (12–281)
Number of patients with Scheuer's stage (I/II/III/IV)	13/5/2/4	88/37/17/13	101/42/19/17
Brinkman index*	400 (0–1575)	0 (0–940)	0 (0–1575)
Number of patients with a history of blood transfusion	3	20	23
Number of patients with a history of drinking	4	7	11
Number of symptomatic patients	4	49	53
Pruritus	1	36	37
Jaundice	2	7	9
Ascites	0	3	3
Varices	2	15	17
Biochemical data			
AST (IU/L)*	59.5 (26–140)	46.5 (16–258)	49 (16–258)
ALT (IU/L)*	72 (17–233)	41 (10–329)	46 (10–329)
ALP (lU/L)*	652 (94–2113)	501.5 (112–2985)	535.5 (94–2985)
*γ*-GTP (IU/L)*	350.5 (75–1349)	164 (13–1004)	173.5 (13–1349)
Alb (g/dL)*	4.2 (2.4–5.1)	4.2 (1.9–5.0)	4.2 (1.9–5.1)
T-Bil (mg/dL)*	0.8 (0.3–3.9)	0.7 (0.1–6.3)	0.7 (0.1–6.3)
IgG (mg/dL)*	1660 (1194–3092)	1676 (778–4532)	1660 (778–4532)
IgM (mg/dL)*	482 (72–1465)	346 (48–1660)	352.5 (48–1660)
Plt (×10^4^/*μ*L)*	24.4 (8.7–58.1)	20.7 (5.3–48.6)	20.9 (5.3–58.1)
PT (%)*	105.0 (78.0–126.5)	100.0 (44.0–139.0)	100.0 (44.0–139.0)
Number of patients with medications (UDCA/BF/UDCA + BF/none)	17/0/7/0	127/1/22/5	144/1/29/5
Number of deaths during follow-up	4	27	31

*Data expressed as median (range); ^†^Data include first year of follow-up.; Alb: albumin; ALP: alkaline phosphatase; ALT: alanine aminotransferase; AST: aspartate aminotransferase; BF: bezafibrate; *γ*-GTP: *γ*-glutamyltranspeptidase; Plt: platelet; PT: prothrombin time; T-Bil: total bilirubin; UDCA: ursodeoxycholic acid.

**Table 2:** Comparison of clinicopathological characteristics between patients who had developed hepatocellular carcinoma (HCC) and those who had not developed HCC at follow-up.

	Overall			Female		
	Patients who had developed HCC at follow-up	Patients who had not developed HCC at follow-up	*P*	Patients who had developed HCC at follow-up	Patients who had not developed HCC at follow-up	*P*
Number of patients	13	166		11	144	
Number of male patients	2	22	0.687	NA	NA	NA
Age of Dx of PBC (years)*	63 (46–77)	56 (22–85)	0.072	63 (46–77)	56 (24–85)	0.049
PBC Stage at Dx of PBC (I/II/III/IV)	2/4/3/4	99/38/16/13	0.004	2/3/3/3	86/34/14/10	0.011
Number of symptomatic patients	8	45	0.022	7	42	0.037
Pruritus	4	33	0.474	4	32	0.282
Jaundice	1	8	0.501	1	6	0.409
Ascites	0	3	1	0	3	1
Varices	5	12	0.003	4	11	0.013
Biochemical data						
AST (IU/L)*	78 (30–231)	46 (16–258)	0.088	77 (30–231)	46 (16–258)	0.15
ALT (IU/L)*	51 (22–319)	45 (10–329)	0.644	47.5 (22–319)	41 (10–329)	0.761
ALP (IU/L)*	402.5 (175–1185)	543.5 (94–2985)	0.314	402.5 (175–1185)	543.5 (112–2985)	0.489
*γ*GTP (IU/L)*	174.5 (16–470)	173.5 (13–1349)	0.285	114 (16–470)	173.5 (13–1004)	0.181
lgG (mg/dL)*	2165 (1030–4532)	1655 (778–3900)	0.021	2165 (1030–4532)	1660 (778–3900)	0.049
lgM (mg/dL)*	405 (136–918)	351.5 (48–1660)	0.364	359.5 (136–918)	336 (48–1660)	0.352
Alb (g/dL)*	3.7 (1.9–4.6)	4.2 (2.0–5.1)	0.016	4.0 (1.9–4.6)	4.2 (2.0–5.0)	0.085
T-Bil (mg/dL)*	0.7 (0.3–2.5)	0.7 (0.1–6.3)	0.925	0.7 (0.3–2.5)	0.7 (0.1–6.3)	0.888
Plt (×10^4^/*μ*L)*	12.4 (5.3–24.4)	21.4 (5.3–58.1)	0.001	12.3 (5.3–21.8)	21.0 (5.3–48.6)	0.001
PT (%)*	96.5 (57.0–117.0)	100.9 (44.0–139.0)	0.204	93.0 (57.0–117.0)	100.9 (44.0–139.0)	0.123
ANA positive	7	106	0.658	6	98	0.579
Brinkman index*	0 (0–400)	0 (0–1575)	0.175	0 (0-0)	0 (0–940)	0.12
Number of patients with a history of blood transfusion	3	20	0.415	3	17	0.285
Number of patients with a history of drinking	0	11	1	0	7	1
Number of patients with a familial history of malignancy	3	61	0.359	2	56	0.261
Number of patients with a familial history of liver disease	1	25	0.764	0	24	0.337
Number of patients with DM	2	17	0.632	2	14	0.317
Number of patients with HT	5	27	0.059	4	22	0.09
Number of patients with HL	3	40	1	3	34	0.725
Number of patients with autoimmune disease	5	53	0.759	5	51	0.528
Sjogren's syndrome	2	24	1	2	24	1
Hashimoto's disease	3	15	0.128	3	15	0.12
Rheumatoid arthritis	1	15	1	1	14	1
Scleroderma	0	15	0.606	0	15	0.602
Systemic lupus erythematosus	0	2	1	0	1	1
BMI*	24.3 (13.8–27.4)	22.6 (14.3–33.1)	0.341	24.3 (15.8–27.4)	22.6 (16.0–32.5)	0.536
Number of patients with medications						
UDCA/BF/UDCA + BF/none	13/0/0/0	131/1/29/5	0.388	11/0/0/0	116/1/22/5	0.512
Number of patients with biochemical response to UDCA and of those with medications						
Responder/non-responder/BF only/no medication (Rotterdam^†^)	11/2/0/0	141/19/1/5	0.911	9/2/0/0	123/15/1/5	0.696
Responder/non-responder/BF only/no medication (Barcelona^†^)	9/4/0/0	80/80/1/5	0.701	7/4/0/0	67/71/1/5	0.816
Responder/non-responder/BF only/no medication (Paris^†^)	12/1/0/0	134/26/1/5	0.832	10/1/0/0	119/19/1/5	0.905
Responder/non-responder/BF only/no medication (Toronto^†^)	11/2/0/0	130/30/1/5	0.914	9/2/0/0	113/25/1/5	0.928
Modalities (US/CT/MRI/US-CT^‡^/US-MRI^‡^/CT-MRI^‡^/US-CT-MRI^‡^)	12/0/0/1/0/0/0	129/10/0/22/3/1/1	0.96	10/0/0/1/0/0/0	114/7/0/18/3/1/1	0.985
HCC screening interval (months)	9 (6–12)	6 (6–12)	0.435	12 (6–12)	6 (6–12)	0.413

*Data expressed as median (range); Alb: albumin; ALP: alkaline phosphatase; AMA: anti-mitochondrial antibody; ANA: antinuclear antibody; ALT: alanine aminotransferase; AST: aspartate aminotransferase; BF: bezafibrate; BMI: body mass index; CT: computer tomography; DM: diabetes mellitus; Dx: diagnosis; *γ*-GTP: *γ*-glutamyltranspeptidase; HCC: hepatocellular carcinoma; HT: hypertension; HL: hyperlipidemia; MRI: magnetic resonance imaging; NA: not applcable; PBC: primary biliary cirrhosis; Plt: platelet; PT: prothrombin time; T-Bil: total bilirubin; UDCA: ursodeoxycholic acid; US: ultrasonography. ^†^Responses were defined as biochemical response to UDCA according to the Barcelona, Paris, Rotterdam, and Toronto definitions. ^‡^These modalities were alternately applied to the patients.

**Table 3:** Standardized incidence ratio (SIR) and standardized mortality ratio (SMR) of HCC in primary biliary cirrhosis.

	Number of incidence of HCC			
	Observed	Expected	SIR	95% Cl
Overall	13 (12)	1.1 (1.0)	11.6 (11.5)	6.2–19.8 (6.0–20.2)
Female	11 (10)	0.5 (0.5)	20.4 (19.8)	10.2–36.5 (9.5–36.4)

	Deaths due to HCC			
	Observed	Expected	SMR	95% Cl

Overall	9	0.9	11.2	5.4–20.6
Female	8	0.4	21.5	9.8–40.7

Including all years of follow up (Excluding experience in first year after diagnosis of primary biliary cirrhosis); CI: confidence interval; HCC: hepatocellular carcinoma; SIR: standardized incidence ratio; SMR: standardized mortality ratio.

**Table 4:** Primary cause of death.

Cause	Male	Female	Overall
	(*n*)	(*n*)	(*n*)
Malignancies	2	11	13
Hepatocellular carcinoma	1	8	9
Others	1	3	4
Lung cancer	1		
Malignant lymphoma		1	
Uterine corpus cancer		1	
Skin cancer		1	
Liver failure (nonmalignant)	1	8	9
Others	1	8	9
Subarachnoid hemorrhage	1		
Heart failure		3	
Pneumonia		1	
Superior mesenteric artery thrombosis		1	
Gastrointestinal bleeding		1	
Sepsis		1	
Acute heart disease		1	

Total	4	27	31

*n*: numbers of patients.

**Table 5:** A risk factor associated with the development of HCC. Adjusted odds ratios derived from the Cox proportional hazards model.

Factor	Overall		Female	
	OR (95% Cl)	*P*	OR (95% Cl)	*P*
Serum albumin levels	0.205 (0.091–0.459)	<0.001	0.228 (0.093–0.557)	0.001

OR: odds ratio; Cl: confidence interval.
